# Eating disorders and sexuality: A quantitative study in a French medically assisted procreation course

**DOI:** 10.1002/brb3.2196

**Published:** 2021-06-17

**Authors:** Marie Audier‐Bourgain, Thierry Baubet, Alexandra Pham‐Scottez, Maurice Corcos, Isabelle Nicolas

**Affiliations:** ^1^ Department of Children and Adolescents Psychopathology Avicenna Hospital General Psychiatry and Addictology Specialized Bobigny France; ^2^ Psychiatric Orientation And Reception Center GHU Paris Psychiatry and Neurosciences Paris Cedex 14 France; ^3^ Department of adolescent and young adult psychiatry Institut Mutualiste Montsouris Paris cedex 14 France

**Keywords:** eating disorder, infertility, medically assisted procreation, sexuality, women

## Abstract

**Objective:**

Among medically assisted procreation (MAP) patients, the prevalence of eating disorders (ED), clinical or subclinical, current or past, is considerable. In addition to metabolic repercussions, the literature suggests that these patients present with more sexual dysfunction, leading to anxiety.

This quantitative research on a population of women consulting a MAP department for unexplained or ovulatory infertility proposed to study the sexuality of this population by comparing the sexuality of patients suffering from or having suffered from an ED to the sexuality of the non‐ED group.

**Method:**

Patients (*n* = 61) filled out the Eating Disorder Examination Questionnaire (EDE‐Q), the Brief Index of Sexual Functioning for Women (BISF‐W), the ED Whole Life Research Questionnaire, the Hospital Anxiety and Depression Scale (HADS) and the Kansas Marital Satisfaction Scale (KMSS).

**Results:**

We found a prevalence of 54% of ED, current or past. Even though these patients reported the same prevalence of sexual intercourse, they had significantly more physical problems (e.g., anorgasmia, vaginismus, headache) affecting their sexuality (*p* = .01) than the non‐ED group, after adjusting for depression. Approximately 10% of the study population reported no intravaginal intercourse during the last month.

**Discussion:**

This study provides evidence for the existence of more sexual dysfunction in patients who have a fertility disorder and have ED or a history of ED. Future research should evaluate the results of psychological or sexological care that may be more suitable for the infertility of these patients.

## Graphical abstract

Our article explores the sexuality of patients suffering from an eating disorders or having a history of eating disorders, and consulting with a diagnosis of ovulatory infertility or unexplained infertility in Medically Assisted Procreation (MAP) departement. The objective was to find out if the use of MAP tehniques could respond to sexual dysfunction within the couple. No difference in the number of vaginal penetrations has been found but sexuality is described as more problematic.

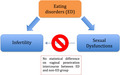

## 1 INTRODUCTION

### 1.1 Epidemiological data

In France, the biomedical agency reported 142,708 uses of assisted reproduction in 2012 compared to 131,736 in 2009. The children, conceived after 23,887 medically assisted procreation (MAP) in 2012, represent 2.9% of all children born to the general population that year, or approximately one child out of 35. After a year of attempting to conceive without contraception, 18% to 24% of couples remain childless. After two years, 8% to 11% of couples are still waiting for a pregnancy (National French Institute of Health and Medical Research, INSERM, 2016). An ovulation disorder accounts for 32% of infertility cases among women (Thonneau et al., 1991). Clinical and para‐clinical investigations will not determine the organic etiology of infertility in 8% to 30% of couples (Ray et al., 2012; Thonneau et al., 1991). Psychological causes can then be mentioned, and among them, eating disorders.

### 1.2 Eating disorders and fertility

Eating disorders (ED) are biologically related to fertility. Leptin, a hormone produced by adipose tissue, plays a central role in reproduction. Its concentration in plasma is directly correlated with the body mass index (BMI) and, more specifically, with the fat mass. A minimal concentration of leptin is necessary for ovulation. This is the biological factor most strongly correlated with amenorrhea in anorexic patients (Bruneau et al., 1999). Amenorrhea occurs after a 10% to 15% loss of ideal body weight (ESHRE Capri Workshop Group, 2006). Regular cycles, reflecting good hormonal functioning, would require a BMI>19 kg $/m^{2}$ but also the absence of weight control behaviors, such as severe dieting, vomiting, or the misuse of laxatives (Abraham, 1998). A study found a high prevalence of menstrual disorders in women with subclinical ED and normal BMI compared to a non‐ED group, matched on BMI (Kreipe et al., 1989).

In the MAP pathways, the prevalence of patients with anorexia nervosa (AN) or bulimia nervosa (BN) is four to five times higher than in the general population (Abraham, 1998; ESHRE Capri Workshop Group, 2006)(Freizinger et al., 2010; Resch et al., 1999; Stewart et al., 1990). High scores are particularly found in patients with no organic cause of infertility, even in the absence of thinness (Bates et al., 1979)(Resch et al., 1999)(Lamas et al., 2006).

These disorders are often difficult to detect by MAP professionals. In one study, 76.4% of participants with ED had not reported this antecedent to their MAP team (Freizinger et al., 2010). Furthermore, infertility treatment is expensive and psychologically laborious. In the ignorance of an ED’s presence, the proposed care might be insufficient or even inappropriate. The psychological problem is not considered, despite the repercussions that it may have during pregnancy and motherhood (Easter et al., 2015; Watson et al., 2013). Indeed, several publications have suggested negative consequences such as depression, attachment disorders, socialization disorders, and conduct disorders in children of mothers affected by eating disorders (Bulik et al., 2005; Haycraft & Blissett, 2010; Lai & Tang, 2008; Leinonen et al., 2003; Zerwas et al., 2012).

### 1.3 Eating disorders and sexuality

In addition to the hormonal impact on libido, many psychoanalytical works describe an avoidance of sexual intercourse, or anxiety about it, among patients with ED. On the other hand, most scientific exploratory studies of ED sexuality present with several biases. First, a sampling bias due to a low number of participants. Second, the metabolic aspect, which must be reasonably considered as one of the causes of a decline in sexual interest, is not taken into account. Third, the evaluation tools available are often reductive and do not allow for the appreciation of all of the aspects involved in this vast question. Nevertheless, they reflect a questioning that has been present for many years.

A high relapse rate and a frequent transition from one type of ED to another suggest they all share a common psychopathology. The most obvious data in the scientific literature are mainly relational difficulties, especially concerning their sexual aspects, with, as a minimum, greater dissatisfaction and anxiety in patients with ED compared to healthy subjects (Carter et al., 2007; Castellini et al., 2012; Gonidakis et al., 2015; Mazzei et al., 2011). Few characteristics specific to each subgroup of ED have emerged (Beerens et al., 2014; Pinheiro et al., 2010).

Many authors have highlighted the central role of body image perturbations in this process. Several authors have also questioned the limited research findings on this topic and have looked for variables that may explain the relationship between ED and sexuality: associations between personality traits, a bad image of the body, and a history of sexual trauma (Wiederman, 1996), correlations between the severity of sexual dysfunction and the severity of body preoccupation in patients with ED (Castellini et al., 2012). Body image preoccupations precede the ED (Rosen, 1996), and constitutes one of the most prevalent residual symptoms (Castellini et al., 2011; Löwe et al., 2001; Ricca et al., 2010; Rosen, 1996; Vall & Wade, 2016). Thus, the whole ED symptomatology is characterized by an extreme silhouette or weight concern, which impairs self‐esteem and affects sex life.

### 1.4 Objectives of our study

Correlations between sexual behavior and EDs, however modest, are present. We make the assumption that the association of rejection of sexual activity with a hormonal disorder can therefore reasonably be raised as the cause of fertility disorders in many patients with EDs, consulting in MAP.

The main objective of this study was to compare the sexuality of women who have or have had an ED with those of women with no previous history of ED, in a MAP department.

To explore this issue, we decided to conduct a quantitative study on the current population consulting the MAP for unexplained infertility and ovulation disorders at the Institut Mutualiste Montsouris, a French hospital.

## 2 MATERIALS AND METHODS

### 2.1 Study design and population

This prospective, single‐center study was conducted in the medical assisted procreation unit of the Mutualiste Montsouris Institute in Paris, France. The patients were recruited from September 2015 through July 2016. After receiving their consent, patients were included in the study if they were women aged from 18 to 42 years, spoke French, and had consulted the MAP for unexplained infertility, primary or secondary, or ovulatory infertility, central or peripheral, before pregnancy.

Women were excluded if they were pregnant or no longer followed in the MAP after completing the questionnaires.

### 2.2 Intervention

After diagnosing an ovulation disorder or unexplained infertility, the MAP medical team informed the patients that they would be contacted shortly for a study. During the consultation, the patient's identifiers and contact information were collected by a member of the staff. The patients were subsequently contacted by the research team to explain the study objectives. If they agreed to participate, they were sent a questionnaire.

The questionnaire included the Eating Disorder Examination Questionnaire (EDE‐Q), the Brief Index of Sexual Functioning for Women (BISF‐W), the Hospital Anxiety and Depression Scale (HADS), and the Kansas Marital Satisfaction Scale (KMSS). These last three questionnaires have been validated in their French versions (Baudelot‐Berrogain et al., 2006; Schumm et al., 1985; Untas et al., 2009). An ED Whole Life Research Questionnaire was also sent, which is used in the epidemiologic research conducted by our research team (Godart et al., 2013).

Subsequently, it was sufficient for them to return it by means of the included prestamped envelope. If they had any questions, the patients could contact the team by mail.

### 2.3 Outcomes

#### 2.3.1 Primary outcome

To screen the ED cohort, we used the Eating Disorder Examination Questionnaire (EDE‐Q) and the ED Whole life Research Questionnaire.

The EDE‐Q is a 36‐item measure of general ED symptoms and features during the last 28 days. It includes four subscales of Restraint, Eating Concern, Shape Concern, and Weight Concern, plus a global score. The EDE‐Q has shown good psychometric properties and the global score has been used as a measure of overall eating psychopathology (Ekeroth et al., 2013).

We used an optimal cut‐off of 1.62 for a BMI less than 18.5 kg/$m^{2}$ (sensitivity and specificity of 0.86), a cut‐off of 2.51 for a BMI between 18.5 and 24.9 kg/$m^{2}$ (sensitivity of 0.89 and specificity of 0.90), and one of 3.15 for a BMI under 25 kg/$m^{2}$(sensitivity and specificity of 0.89) (Rø et al., 2015).

The ED Whole Life Research Questionnaire was used to identify any history of AN, BN, or other specified eating or feeding disorders (OSFED).

To compare the sexuality of patients who have or have had an ED with those of patients with no previous history of ED by using the Brief Index of Sexual Functioning for Women (BISF‐W).

The BISF‐W (Taylor et al., 1994) is a 22‐question (49‐item), a multidimensional, self‐reported instrument for qualitatively and quantitatively assessing female sexual function and satisfaction levels, through seven Dimension (D) scores (D1: Thoughts/Desire; D2: Arousal; D3: Frequency of Sexual Activity; D4: Receptivity/Initiation; D5: Pleasure/Orgasm; D6: Relationship Satisfaction; D7: Problems Affecting Sexual Function =bleeding, irritation, lack of lubrication, painful penetration, difficulty having an orgasm, vaginismus, urine leakage, headache, and vaginal infection). D1, D2, and D5 reflect Kaplan's three‐phase model for sexual response (Hawton, 1980), considered to be a conceptual model for assessing female sexual dysfunction (Derogatis & Conklin‐Powers, 1998). D2 combines subjective perceptions of sexual arousal with the sensation of sexual inhibition and anxiety. D3 rates the quality and frequency of sexual activity. D4 rates the individual propensities to transform sexual desire and fantasies into sexual behavior. D6 and D7 qualitatively rate the emotional context in the sexual relationship and several problems that could affect sexual function.

#### 2.3.2 Secondary outcomes

##### Anxio‐depressive disorders and marital satisfaction were adjustment variables to take into account

‐ Anxio‐depressive comorbidities were explored with the Hospital Anxiety and Depression Scale (HADS). The Hospital Anxiety and Depression Scale (HADS) is a frequently used self‐rating scale developed to assess psychological distress in nonpsychiatric patients. It consists of two subscales, Anxiety and Depression (Zigmond & Snaith, 1983). A score of 11 or more on the HADS defines a depressive or anxious syndrome.
‐ Conjugal satisfaction, which allowed the best interpretation of the results of the BISF‐W, was assessed by the KMMS. The KMMS is a 3‐item self‐reported instrument designed to measure marital quality (Nichols et al., 1983). Items are rated on a 7‐point Likert scale, ranging from one (extremely dissatisfied) to seven (extremely satisfied). The total score ranges from 3 to 21, with high scores meaning better marital quality.

### 2.4 Statistical analysis

The minimum number of subjects required in each group was 28 (alpha risk of 0.05, power of 90%, unilateral comparison of average BISF‐W scores between the two groups, with the hypothesis of a normal distribution in each group, and a standard deviation of the differences in each pair of subjects equal to 5).

#### 2.4.1 Univariate analysis

Qualitative variables were compared using the Chi‐square test or Fisher's exact test, as appropriate. Quantitative variables were compared using Student's *t*‐test. A point graph and Pearson correlations were used to find the link between two quantitative variables.

#### 2.4.2 Multivariate analysis

A linear regression model was performed to identify variables that significantly influenced the primary outcome.

### 2.5 Legal and ethical aspects

This study was approved by the Committee for the Protection of Persons (CPP) of Cochin (Ile de France III) on 22 September 2015. Each subject was informed of the objectives and the mode of conducting the research, as well as the nonbinding nature of their participation. An information sheet and a consent form for participation in biomedical research were provided to them prior to inclusion. If ED or depressive symptoms were detected, an adapted follow‐up was proposed to the participants.

## 3 RESULTS

### 3.1 Description of the population

**FIGURE 1:**
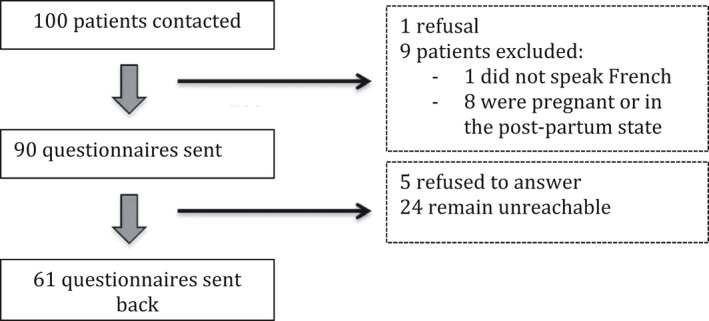
Flow Chart

All patients who agreed to participate in the study were included consecutively (Figure 1). Among the 61 patients who filled out the questionnaires, 44 were diagnosed with unexplained infertility (72.1%) and 17 had an ovulation disorder (9%). Most of them were of a high socioeconomic level, representing the general patients of the Mutualiste Montsouris Institute. The mean infertility duration was over three years (40 months, ± 18.7) for patients who had used contraceptives prior to deciding to have a child (*n* = 48, 78.7%). The mean BMI was 22.5 kg/$m^{2}$ (± 4.3) (Table 1). Eight patients had a BMI less than 18.5 kg/$m^{2}$ (13.1%). Six of these eight patients had a history of EDs. One of them had a current ED.

**TABLE 1:** Baseline characteristics

	Total (*n* = 61)	ED (*n* = 33)	Non‐ED group (*n* = 28)
Cohabitation length – years (± *SD*; min – max values)	8.5 (± 3.3; 2 – 20)	8.1 (± 3.2; 2 – 16)	9.0 (± 3.3; 5 – 20)
Mean age ‐ years (±*SD*; min – max values)	33.7 (± 3.9; 24 – 42)	33.2 (± 4.0; 24 – 41)	34.4 (± 3.8; 25 – 42)
Mean time after stopping use of contraception – months (±*SD*; min – max values)	40.1 (±18.6; 0 – 81)*	41.6 (± 20.9; 0 – 81)**	37.5 (± 14.3; 0 – 60)***
Mean current BMI ‐ kg/$m^{2}$ (±*SD*; min – max values)	22.5 (±4.3;16.4 – 33.2)	22.4 (± 4.6; 16.8 – 33.2)	22.5 (± 4.1; 16.4 – 30.5)
Mean ideal BMI ‐ kg/$m^{2}$ (±*SD*; min – max values)	20.7 (±2.7;16.3– 27.0)	20.4 (± 2.7; 16.3 – 27.0)	20.9 (± 2.6; 17. – 26.0)

Abbreviation: *SD*, Standard deviation.

* *n* = 48;

** *n* = 30;

*** *n* = 18.

### 3.2 Frequency of eating disorders and anxio‐depressive syndrome

**FIGURE 2:**
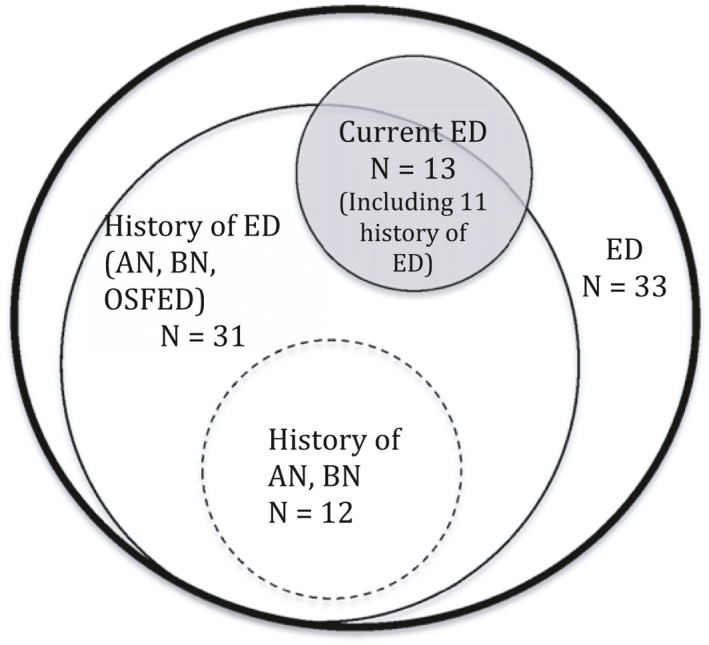
Distribution of Patients with Eating Disorder (ED), current ED or past ED. (ED, Eating disorders history past or current; Current ED, eating disorders in the last 28 days; History Of ED, whole life history of ED; History of AN, BN, whole life history of anorexia nervosa or bulimia)

After EDE‐Q and whole‐life ED questionnaire analysis, four groups were constituted (Figure 2):

‐ ED group (*n* = 33, 54.1%): patients with a present or past clinical or subclinical ED.
‐ Current ED group (*n* = 13, 21.3%): patients with a current AN, BN, or other specified feeding or eating disorders (OSFED) diagnosis.
‐ History of a clinical ED group (*n* = 12, 19.7%): grouping patients with a diagnosis of AN, or BN in their entire lifetime.

Five patients (8.2%) had probably a depressive disorder and 13 (21.3%) had probably an anxiety disorder. In our study, past or current ED and depression were noted to be significantly correlated (*p* >.2).

### 3.3 Sexuality of the global study population and comparison of sexual function in women with or without ED compared to the non‐ED group

Some items are not taken into account in the BISF‐W overall score(Table 2):

‐ Modifications of activity, interest, excitement, and sexual satisfaction.
‐ Homosexual desires and experiences.

**TABLE 2:** Dimension scores of BISF‐W in the global population

	thoughts/ desires	arousal	frequency of sexual activity	receptivity, initiation	pleasure, orgasm	relationship satisfaction	problems affecting sexual function
Means	5.3	6.9	3.6	9.3	5.0	9.0	3.6
Standard deviation	2.5	2.3	1.9	3.2	2.1	2.3	1.9
Minimum	0	0	0	0	0	0	0
Maximum	13	11	7	15	9	12	9

In terms of activity, 30 patients (50.8%) reported having a markedly or slightly decreased sexual activity, 20 patients (32.7%) had a loss of interest, 19 patients (31.1%) had a loss of excitement and 13 patients (21.3%) considered themselves sexually less satisfied. Eleven patients (18%) mentioned some homosexual desires, but only two patients (3.3%) reported some homosexual experiences. There were no significant differences in these variables between the ED and non‐ED groups.

In all BISF‐W categories, the current ED groups reported more problems related to sexuality than the non‐ED group (mean =2.74 versus 4.40, *p* =.01). This significant difference was observed in both the previous ED group (mean =3.05 versus 4.10, *p* =.033) and the current ED group (mean =3.33 versus 4.51; *p* =.049).

The thought and desire scores were higher in the current ED group than in the non‐ED group (mean =6.99 versus 4.96, *p* =.023). There were no significant differences between the two groups in the other categories. Depressed patients had a lower overall score than the other patients (mean =25.68 versus 36.44, *p* =.046) and reported more problems (mean =5.53 versus 3.41, *p* =.01). After adjusting for depression, the results obtained for the ED group were still related to the BISF‐W problem score. Sexuality for patients with or suffering from ED is therefore significantly more problematic. On the other hand, patients currently suffering from ED had more desire and sexual thoughts, without more sexual activity.

The frequency of intravaginal intercourse was calculated by data from the BISF‐W (Table 3). Six patients (9.8%) reported no intravaginal intercourse for one month. Of these six patients, three described an unchanged sexual activity, suggesting that the absence of sexual intercourse was common for the couple. The other three (*n* = 3) strangely reported having no sexual partner, while they were part of a couple and consulting for MAP. Three (50%) of these six patients had or did have an ED. To make comparisons, we classified the frequency of intercourse into two classes: not procreative, less than or equal to one time per month (*n* = 10, 16.39%), and procreative, greater than or equal to two times per month (*n* = 51, 83.6%). After analysis, there was a trend in the patient group with a history of AN or BN with a low frequency of sex (40% versus 15.7%, *p* =.096). We did not find any link between the frequency of sex and depression.

**TABLE 3:** Frequency of intra vaginal intercourse depending on the presence of a clinical or subclinical ED, current or past, in the last month

		Diagnostic	
		Non‐ED group (*n* = 28)	ED (*n* = 33)
Number of vaginal penetrations in the last month	Never	3 (11%)	3 (9%)
	Once a month	2 (7%)	2 (6%)
	2 to 3 times a month	3 (11%)	5 (15%)
	Once a week	4 (14%)	5 (15%)
	2 to 3 times a week	14 (50%)	18 (54.5%)
	Once a day	1 (3.5%)	0
	More than 1 time a day	1 (3.5%)	0

### 3.4 Conjugal relationship

Based on the KMSS scale from 0 to 7, the average rating of marital satisfaction was 5.9 (± 1.13, min =2.7, max =7) and 20 patients (32.8%) reported having a fully satisfactory marital relationship. These results were significantly related to the BISF‐W sexual satisfaction score (*p* =.007).

## 4 DISCUSSION

Among our recruitment of MAP consultants for unexplained infertility or ovulation disorders, 54.1% of patients had suffered from or still suffered from a clinical or subclinical ED (OSFED), 13% still suffered from an ED, and 50.8% had a history of ED, while 19.7% had a history of a clinical ED: AN or BN.

In the study population, 50.8% of the patients reported a decrease in sexual activity, 32.7% a decrease in interest, and 21.3% a decrease in sexual satisfaction. Nevertheless, in the general population, 10% of patients reported not having sexual activity, half of whom had not had any changes in sexual activity, suggesting a lack of normal sexual relations within the couple. There was no significant difference between the ED group and the non‐ED group in sexual frequency. On the other hand, patients suffering from or having suffered from an ED reported more problems affecting sexual function (BISFW D7 item) than that of the non‐ED subjects but also more sexual desire. In the global population of our sample, 8.2% of patients had a depressive syndrome and 21.3% had anxiety.

Our study did not find any clear difference between the sexual behavior of patients affected by ED and that of the non‐ED group, including the frequency of sexual intercourse. We found only a trend for patients with an AN or BN episode characterized in the past. It also seems important to point out that desiring a child and participating in the MAP process also impact sexuality (Bokaie et al., 2015; Carvalho & Nobre, 2010; Czyżkowska et al., 2016; Winkelman et al., 2016).

The BISF‐W scores correspond to the averages obtained in the Francophone validation study of the general population of nulli or primiparous women (mean Desire score =4.4 ± 2.2, Excitation =6.5 ± 2.4 Frequency =3.4 ± 2.0 Initiative =9.1 ± 3.6 Pleasure =4.7 ± 2.0 Satisfaction =8, 9 ± 2.3, Problems =3.6 ± 1.9)(Baudelot‐Berrogain et al., 2006). The results obtained on the ED patients’ sexuality are supported by the scientific literature. Indeed, this study found some problems related to sexuality among the patients suffering from or having suffered from an ED, compared to the non‐ED group (anxiety, dissatisfaction, organic manifestations). However, it did not find avoidance of intercourse, except for women with a low BMI, which is probably due to a hormonal impact (Castellini et al., 2016; Mazzei et al., 2011; Pinheiro et al., 2010). Most of these studies used the female sexual function index for the evaluation of sexuality. We chose the BISF‐W because the FSFI items are mainly about physical problems during sexual intercourse, and neither the intercourse frequency nor the intercourse quality is taken into account. The scores obtained in the D1 section of the BISF‐W (Thoughts/desire), relating to sexual thoughts, fantasies, and erotic dreams, in ED patients, are original and could be related to psychoanalytic theories of internalized excitement (Corcos, 2010; Haag, 1984; Kestemberg et al., 1989).

The concepts of pleasure and orgasm seemed to be confusing for several of our patients, with, for example, an orgasmic response score for sensual kisses (27.8%), which seems unlikely. This is not surprising given the prevalence of women who have never had an orgasm (5%, according to the French Public Opinion Institute, IFOP, 2016). Nevertheless, it reveals an ever‐present lack of awareness of bodily responses during sexual intercourse, and suggests a lack of sex education.

Moreover, we questioned a large number of totally satisfactory marital relationships (maximal score for 32.8% patients). We observed high scores on the BISF‐W for some of these patients who declared they were totally satisfied, questioning the authenticity of the answers to the marital quality questionnaire. The patients may have been afraid of a lack of confidentiality in their responses. On the other hand, in a more general process, we can question the role of denial in these patients and their desire to conform to a multilevel performance model, conveyed by the mass media.

The frequency of past or current ED in our cohort is much higher than that found in the literature, where the highest incidence was 20.7% (Freizinger et al., 2010). This difference could be explained by the recent changes in the criteria for a diagnosis of AN in the DSM5, including the withdrawal of amenorrhea from the diagnostic criteria. This increase in the prevalence of AN due to changes in the DSM5 criteria was also found in a recent epidemiological study (Ernst et al., 2017).

Only 8.2% of patients had the severe depressive syndrome, approaching the frequency observed in the general population (Lépine et al., 2005). In contrast, 21.3% of patients had a known anxiety disorder, which is 10 times higher than the prevalence in the general population (Lépine et al., 2005). No link was found between anxiety or depressive syndrome and the presence of an actual episode or history of ED. We believe that the absence of an observed link between depression and ED is due to the study population. Indeed, patients consulting MAP are more likely, due to the psychological impact of the fertility course, to develop an anxio‐depressive syndrome (Boivin et al., 2011). Thus, the population of our sample is, on the whole, more likely to develop a depressive or anxiety syndrome.

### 4.1 Strengths and limitations

Our study explored the sexuality of patients suffering from or having suffered from eating disorders, in a context of a desire for children and a follow‐up in a medically assisted procreation program, compared to that of non‐ED subjects, in the same context. This study is the first to our knowledge exploring the sexuality of these patients in this very particular context. Indeed, while several studies have examined the sexuality of patients with ED, none of them had dealt with the reproductive dimension of sexual function. In particular, this highlighted the choice of some patients to turn to the techniques of MAP to conceive, and thus to overcome their sexual dysfunctions.

However, our research has some limitations: it is a single‐center study, based on self‐assessment questionnaires. Moreover, the low numbers forced us to collect categories in order to obtain larger groups, allowing for comparisons to be made. Thus, for our main objective, we had to make two groups of sexual intercourse frequencies (less than one intravaginal relationship per month or more than two). The recommendations of the MAP are two to three sexual intercourses per week with vaginal penetration. Group 2: "conducive to procreation" is therefore not adequate in following the current recommendations. Nevertheless, it seems to us unrepresentative to group together in the group "not conducive to procreation" women having no intercourse per month and those having between two and three. On the other hand, we started from the principle that vaginal penetration was equivalent to intravaginal ejaculation, which is not necessarily the case.

The questionnaire did not sufficiently explore questions about a disorder of body image. These questions could be developed in the future and could be a key point linking sexual dysfunction and ED. Indeed, we think that the disturbance of the body image, in that it is the result of a lack of identity construction, is the common point between these two pathologies, EDs (Fairburn & Harrison, 2003; Gutiérrez & Carrera, 2016) and sexual dysfunction (Ackard et al., 2000; Morgan et al., 1995; Robbins & Reissing, 2017; Tolosa‐Sola et al., 2017), leading, each independently of the other, to fertility disorders (Figure 2). It is important to take into account that women in this type of care course are often under hormonal stimulation. However, this type of treatment has a non‐negligible body image impact. Thus, the large proportion of patients preoccupied with their body image, with a desire for weight loss, can only be partly explained by these conditions.

We have chosen, for ethical reasons, not to look for a history of sexual abuse. Indeed, any disturbing results would have imposed the necessity of a proposal of care that we were not able to provide. However, the presence of a history of sexual trauma in ED patients is frequent and also impacts the sexuality of these patients (Castellini et al., 2013).

## 5 CONCLUSION AND IMPLICATIONS

Our study is the first to explore the sexuality of patients suffering from or having suffered from an ED and desiring a child, seeking assisted procreation. In this population of patients using MAP techniques, there is no avoidance of sexuality, as it can be classically described. On the other hand, their sexuality is described as more problematic, with a probable psychological impact at this level within the couple's relationship, which can cause a feeling of malaise in these patients.

Currently, in France, social security does not reimburse consultations with a psychologist, but most MAP units have a psychologist to support couples who explicitly express a desire to consult one. This recourse is, however, quite rare. We believe that most couples are afraid to seek this care for fear of delaying MAP interventions or seeming to be too vulnerable. A systematic proposal to the couples of support in space independent of the MAP service could be valuable.

In the future, we would like to continue to explore the psychological difficulties encountered by patients, and more generally by couples, who are consulting for MAP. The interventions currently proposed, in terms of psychotherapeutic or sexological care, although they have the merit of existing, appear in the eyes of these patients to be insufficient. Other studies will eventually suggest new ways of care and support, in order to further personalize the therapeutic proposals.

## CONFLICTS OF INTEREST

No author of this article has any conflict of interest, financial or otherwise, related to the submitted work.

### PEER REVIEW

The peer review history for this article is available at https://publons.com/publon/10.1002/brb3.2196.

## Data Availability

The data that support the findings of this study are available from the corresponding authors upon reasonable request.
